# A democracy built on communicative action: Bahá'í political practice as a prefigurative resource for institutional effectiveness, accountability, and inclusivity

**DOI:** 10.3389/fsoc.2023.965428

**Published:** 2023-07-26

**Authors:** Michael Sabet

**Affiliations:** Department of Political Science, University of Toronto, Toronto, ON, Canada

**Keywords:** institutions, Habermas, communicative action, prefigurative politics, deliberative democracy, Bahá'í, democratic theory, comparative political theory

## Abstract

Goal 16 of the UN sustainable development goals, which calls on the global community to “build effective, accountable and inclusive institutions at all levels,” can be conceptualized as aiming at fostering communicative action, a concept developed by Jürgen Habermas to describe a mode for coordinating society grounded in deliberation. However, Habermas simultaneously provides an account of the structural transformation of the public sphere that suggests a hard limit on the capacity of mainstream capitalist liberal democracies to foster genuine communicative action in the relationships between institutions, individuals and communities. This paper therefore argues for the critical role of prefigurative politics, in which communities strive to internally embody desired socio-political forms rather than focusing on changing the wider socio-political order, as a vital resource for generating examples to inform institutional progress. The prefigurative example of the Baha'i community demonstrates norms and practices that may illustrate a path out of the dynamic Habermas identifies of system colonizing lifeworld, by fostering and protecting communicative action as the mode of social coordination. The form of communicative action found in the Baha'i community is situated in a context of a *telic-*organic model of relationships between individuals, communities and institutions. The paper contrasts the conceptual underpinnings of this model with individualistic conceptions of human nature that are argued to undermine liberal democracy's capacity for communicative action. At the core of communicative action within a Baha'i context is a distinctive model of deliberation, known within the community as “consultation”. The paper argues that *rational-critical consultation* can offer a vital nuance to Habermas' ideal of communicative action as *rational-critical debate* in the public sphere. The formal democratic structures and processes of the Bahá'í community are also explored as an institutional example that arguably meets the challenge of Goal 16. The paper concludes with initial reflections on a process by which the prefigurative example of a Baha'i model might be brought to bear on institutional performance in wider society.

## 1. Introduction

Goal 16 of the UN sustainable development goals calls on the global community to “build effective, accountable and inclusive institutions at all levels” [United Nations, (n.d.)]. In this paper, I take the invitation to “build” institutions at face value, by exploring a particular prefigurative political tradition—the global Bahá'í community—as a potential source of insight for innovation in the structure of political institutions and in their relationship with the communities they serve.

To argue the relevance of prefiguration in general, and of the Bahá'í experience specifically, I draw on two interconnected strands of Jürgen Habermas' thought. Taking Habermas' concept of communicative action as a standard by which to assess the democratic vitality of a socio-political order, I use his account of the structural transformation of the public sphere to argue that, while the reform and improvement of existing institutions is undoubtedly necessary, it cannot be sufficient to meet Goal 16. I then argue that the structural forces Habermas accentuates were historically buttressed by, and in turn selected for, certain crucial ideas centering on individualism that contributed to the erosion of the public sphere and the sidelining of communicative action in modern politics. Next, I argue that prefigurative communities, which aim to create social change by internally embodying desired socio-political forms rather than focusing on changing the wider socio-political order, can—to the extent that they insulate themselves from the forces driving the structural transformation of the public sphere, and reject the individualistic ideas that underpin it—both accentuate the contingency of our current institutional arrangements, and generate examples to inform radical institutional progress.

This sets up the prefigurative example of the Bahá'í community, which provides an alternative conceptualization of the nature of, and organic relationships between, individuals, communities, and institutions. Its resulting distinctive set of norms and practices may illustrate a path out of the Habermasian dynamic of system colonizing lifeworld by fostering communicative action as the primary means of social coordination. At the core of communicative action within a Bahá'í context is a distinctive model of deliberation, known within the community as “consultation”; the paper suggests that *rational-critical consultation* can offer a vital nuance to Habermas' ideal of communicative action as *rational-critical debate* in the public sphere. The discussion moves from the conceptual underpinnings of a Bahá'í model to their expression in social norms, community dynamics, and formal institutional arrangements, which I contrast with the features of liberal democracies that Habermas posits as disintegrative to the public sphere.

The paper concludes with some reflections on a process by which the prefigurative example of a Bahá'í model might be brought to bear on institutional performance in wider society. While the primary lens of the paper is prefigurative politics, which can encompass both religious and non-religious movements, the conclusion does consider what it might mean for a specifically religious community to serve as a resource for institutional change. While a full exploration of this question is beyond the scope of this paper, I briefly suggest, drawing again on Habermas in his refinement of Rawls' concept of public reason, that the foundational concepts on which the distinctive Bahá'í institutional forms and practices are built can, in fact, be “translated” into terms that can make them comprehensible, and potentially acceptable, to a wider audience.

## 2. Habermas' framework

### 2.1. Communicative action and the transformation of the public sphere

Habermas' social theory centers on the public sphere as the core locus of democracy, the site (potentially) of communicative action—in contradistinction to the state and the market, which operate on the non-discursive logics of bureaucratic power and money respectively (Habermas, 1989). Communicative action is a means for coordinating human affairs based on the application of reason. For Habermas, the ideal of communicative action is one of rational-critical debate, in which participants of diverse social backgrounds discuss together, and are swayed by the “unforced force of the better argument” rather than a speaker's status (Habermas, 1996; p. 306). While Habermas' later work focuses on a transhistorical analysis of communicative action, his earlier scholarship, which traced the historical trajectory of the public sphere, provides crucial insights into the limitations of institutions in capitalist liberal democracies, and is a useful resource for thinking about the parameters of any plausible route toward the progress described in Goal 16.

Habermas' historical argument is most completely articulated in *The Structural Transformation of the Public Sphere* (1989). As sites of critical discussion—coffee houses, salons, and journals, where the literate bourgeoisie met on grounds that diminished the importance of social status—began to shift their attention to political matters, it became possible to imagine social consensus: a public will emerging out of rational-critical debate within this nascent public sphere. The public sphere never achieved its full promise as a site of generative rational-critical debate, but its peak in this regard occurred early in its history, when it was still effectively the domain of the bourgeoisie. The story of its subsequent decline can be told in terms of the rise of mass enfranchisement, the dynamics of capitalism, and the growth of mass media. As more and more groups, of unequal social status, entered the public sphere, the Kantian ideal of the collective exercise of reason leading to a general will was replaced with an ideal of compromising between irreconcilable interests through fair negotiation. The public necessarily delegated this specialized negotiation function to “private bureaucracies, special-interest associations, parties, and public administration,” while its own role was reduced to periodic “acclamation”—voting on platforms produced by these specialists (Habermas, 1989; p. 176). Simultaneously, the economic system of capitalist societies increasingly de-politicized the public sphere by replacing its culture of critical discourse with a culture of consumption.

Both of these disintegrative forces—through which the state and the market colonized the public sphere through non-communicative power and money respectively—were exacerbated by the development of mass media, through which political and economic actors sought to create and mobilize consumers of their respective goods. The public sphere became fragmented, as both political and economic actors reached out to specific constituencies, rather than attempting to engage the public as a whole. Political and economic advertising, which casts its recipient in a passive role, further displaced rational-critical debate in the public sphere.

Habermas' view of modernity is by no means wholly negative: he highlights the remarkable advance that the capacity for communicative action represents historically, while being clear-eyed about the structural forces that have tended to fetter it. However, the historical analysis in *Structural Transformation* does suggest the inevitability—and essential irreversibility—of that fettering. Habermas' work has of course attracted enormous commentary; I do not attempt to review it in this paper, whose goal is not to critique Habermas but to use his framework to shed light on the challenge of meeting Goal 16. What will instead be useful is to briefly review Habermas' own sense of the bounded possibilities for improvement, before considering whether prefiguration can help us realistically envision other possible futures.

### 2.2. Colonization of system by lifeworld

From one perspective, Habermas' story explains how the “system”, i.e., bureaucratic and market procedures, colonizes the “lifeworld,” our noninstitutionalized sphere of being in society amongst family, friends, voluntary associations, etc., as well as the public sphere (Habermas, 1987). The system roots out communicative action through the application of the non-communicative forces of power and money, which come to increasingly coordinate human life.

Crucially, in Habermas' view, the split between lifeworld and system cannot be overcome: the historical trajectory is toward greater integration and complexification of the system, which could only be undone through some catastrophic (and undesirable) collapse. But we can distinguish here between system as simply the institutional realm of modern societies, and system as a non-discursive *approach* to coordination. Can we retain the functions and capacities of, say, the state, while making its institutions meaningfully democratic, accountable and inclusive?

Habermas suggests a partial solution through increased democratic accountability within institutions like political parties. This is a limited, non-ideal solution: it cannot re-integrate the public in a cohesive whole, but simply tries to make the institutions that market themselves to specific fragments of the public more internally democratic. At best, such a solution might allow the negotiation between the institutional representatives of these fragments to be better informed by their constituents' actual views; it cannot restore rational-critical debate to the public sphere as a whole, and thus cannot contribute to true public will formation.

The extent to which Habermas himself holds out hope for even this limited solution is ambiguous. Certainly, Habermas has not suggested that there have been great strides in revitalizing the public sphere since the initial publication of *Structural Transformation* in 1962. Perhaps as a result of the poor prospects on this front, his focus, as noted earlier, has been less on the historical trajectory of the public sphere than on the conceptual requirements of communicative action, conceived of as an evolving capacity rooted in human reason. Yet the practical problem remains: how—given the dynamics of lifeworld and system described above—can communicative action be reliably and sustainably fostered within the liberal democratic state?

Habermas' later writings on communicative action have made great contributions to the literature on deliberative democracy, which offers a talk-centric model of democracy as an alternative, or supplement, to the dominant vote-centric model. On its face, deliberative democracy appears to be a democracy that relies on, and thus helps to restore, communicative action. Where vote-centric democracy treats citizen preferences as essentially fixed, deliberative democracy considers preferences malleable and responsive to a process of reasoning with others (Chambers, 2003; Young, 2004). Deliberative theory argues that people can and should play a more active role in their own governance by reasoning together about policy and taking ownership of the outcomes of such reasoning (Mansbridge, 1983; Thompson and Gutmann, 2004; Neblo, 2015).

However, Habermas' historical analysis in *Structural Transformation* suggests that deliberative democracy, in spite of being supported by a sophisticated literature and a wealth of experiments and experiences around the globe, will continue to live in the shadow of vote-centric democracy, whose role as the tether between the people and their political institutions in liberal democratic society cannot easily be supplanted. While the system colonizes the lifeworld, it is also rooted in its soil; to the extent that it has sterilized that soil of communicative action, it cannot be now be re-infused by it. Further, in such a reality, the prospect for growing deliberative institutions from the ground of the lifeworld are bleak: the soil is arguably too sterile. An enthusiastic political scientist, think tank, or activist organization might create a deliberative *space*, but creating a *culture* of deliberation will feel like swimming upstream.

## 3. Concept of human nature and impacts on communicative action

Before considering how the prefigurative paradigm may help address this structural issue, it is vital to highlight the importance of human imagination and self-perception in shaping the public sphere. A deliberative political culture must be rooted in its members' subjective self-understanding, drawn from shared cultural resources, as people who deliberate (see Habermas, 1996). The structural forces Habermas highlights contribute to limiting the extent to which such a self-understanding can take hold: as norm-free commercial transactions and state-imposed administrative structures come to replace intersubjective relationships in people's lives, the scope for individuals to exercise their capacity to intersubjectively negotiate mutual understanding shrinks (Habermas, 1984). But the structural account risks concealing the importance of certain *ideas*, centering on a kind of individualism, that have also contributed to constraining our self-understanding, not least by helping to drive the contingent trajectory of the structural transformation that Habermas focuses on.

I propose that two, at least, of the critical processes in Habermas' account of the structural transformation of the public sphere—the expansion of the franchise, and the rise of mass communication—contributed to the disintegration of the public sphere because of *how* they were deployed in line with specific ideas. In Habermas' account, the enlargement and diversification of the voting public led to a shift in liberal theory away from belief in the possibility of consensus and public will and toward acceptance of irreducible difference, which it would be the function of politics to negotiate. But this “resignation before the inability to resolve rationally the competition of interests in the public sphere” (Habermas, 1989; p. 135) arguably resulted from the increasingly evident effects of a particular, contingent view of the human being that was already baked into liberal theory.

This view can be traced to pre-liberal thinkers such as Machiavelli, who critiqued the traditional placement of the education and perfection of the individual at the center of political theory. Machiavelli asked, not how a ruler or system could be just, but how a ruler might maintain his rule over people who were selfish, conniving, potentially violent, and—crucially—likely to remain so. Later, liberal thinkers, even those such as JS Mill who believed that humans can recognize and adopt better ways of living, held that the encouragement of virtue was not the concern of politics, which was instead to be the arena in which individuals were given equal opportunity to advance their interests, determined by their own reason. The individualistic framing of this concept of reason contributed to a limited perspective on the possibilities of collective, deliberative rationality; even Mill's marketplace of ideas frames the individual as essentially a consumer of, rather than a deliberator about, ideas (Mill, 2001). Given this view of the individual, it was only prudent and reasonable to design government to depend as little as possible on individuals' capacities to deliberate, cooperate, or otherwise subordinate their irreducible self-interest. Thus, Kant held that an effective republican constitution must harness the people's conflicting selfish desires in such a way as to create public order, making good government possible even for a “nation of devils” (Kant, 2006; p. 90). Similarly, Madison wrote that government is “the greatest of all reflections on human nature. If men were angels, no government would be necessary” (Madison, 2009; p. 120). The role of the state remains essentially Hobbesian—preventing a slide into the chaos that results when people follow their natural inclination to self-interest. The balance point between chaos and tyranny is struck by a minimal virtue approach to government: as Mill puts it, the state's interference should cease at the point where “a person's conduct affects the interests of no persons besides himself” (Mill, 2001; p. 69).

The concept of interests plays a crucial role in this story about the individual, whose goal in politics is conceived of as advancing private, rationally-determined interests, often read as economic interests. Thus, the conception of the human being that animates the capitalist economic model also ends up achieving hegemonic status in liberal democracy: the human being is essentially a rational self-interested actor. Our democratic culture similarly ends up conforming to the broader “culture of contest” that permeates our socio-political-economic life (Karlberg, 2004).

None of this is to suggest that the rational self-interested model of the human being was, or is today, the only one posited in liberal theory or in modern democratic societies. The point is that it was the account of human nature that was selected for by political and economic structures—structures that were themselves designed in keeping with this account. The relationship between ideas and structure is reciprocal (see Giddens, 1984), and there are thus two complementary stories that can be told about the transformation of the public sphere. Habermas' structural story, focusing on the increasing integration of the system, characterizes the marginalization of communicative action in the public sphere as inevitable. The ideational story which I am highlighting, however, suggests that the view of human nature created a self-fulfilling prophecy. Expecting people to be individualistic and self-interested, we structure the world in such a way as to draw out these qualities.^1^ It is no surprise that vote-tallying, rather than deliberation, is the core democratic practice: why provide spaces, mechanisms, and resources for the people to deliberate if they can identify their self-interest by their own unaided reason? Their views will not change with deliberation, so all that is left is negotiation, which will be the purview of the system. The individualistic model of the human being has thus undermined the prospect for meaningful communicative action from the start, and the dominant political processes of liberal democracy come to rely on, and select for, atomistic, non-deliberative attitudes and behaviors in the general public.

It is similarly no surprise that modern political campaigns are designed less to convince voters than to mobilize those who already identify with the party (Habermas, 1989; p. 203-4, Achen and Bartels, 2016). While Habermas explains this focus on mobilization in terms of structural forces—the challenges posed to deliberation by mass enfranchisement, and the publicity enabled by mass communication—it can also be seen as a logical outcome of the view of human nature outlined above. From this perspective, the move from rational-critical debate in the public sphere, to negotiation between institutional actors as the prime site of politics is not an inevitable consequence of expanding the franchise beyond the bourgeoisie. While it is true that a far wider range of interests would be represented as the franchise expanded, this only poses a fundamental problem for rational-critical debate if we accept that differing interests preclude consensus (or, failing consensus, uncoerced unified action following deliberation, as suggested in Bahá'í prefigurative practice; see below).

The dynamic that Habermas places in historical context in Structural Transformation can thus be explained as arising out of the confluence of both the structural forces he describes *and* the preponderating influence of a particular conception of the human being. If the goal of wider and wider enfranchisement is advanced within a paradigm of irreducible self-interest, then the diversity it creates arguably must result in the shift from the rich participation of deliberation to the comparatively thin democracy of negotiation by institutional actors—a democracy that sets a hard limit on meaningful institutional inclusivity and accountability. If there is to be any prospect of negotiating the diversity inherent in modern pluralistic democracies through communicative action instead, this paradigm must be challenged. Prefigurative politics suggests how this can be done.

## 4. The utility of prefigurative examples

Habermas arguably underplays the possibilities inherent in the agency that individuals bring to the deliberate reshaping of the public sphere, in particular through social movements (Calhoun, 1992); this is an oversight that Habermas has sought to rectify in more recent work. However, while social movements can doubtless create counter-currents to the prevailing influences of power and money in the public sphere, they must still swim against the powerful structural tides that Habermas identifies. Indeed, most social movements, to the extent they do not seriously challenge or question the overall logic of state or market, cannot mount a serious resistance to the colonizing tendency of the system (Palmer, 2018, p. 34). Even those elements in civil society within liberal democracies that do hold out some vision of an alternative socio-political order will often operate to achieve this vision within the parameters defined by the dominant system (including by adopting adversarial methodologies—partisanship, protest, etc.—that, even while promising to channel opposition to power, rely upon and reinforce its foundational premises; see Karlberg, 2010).

This suggests that Habermas is not wrong to be skeptical of the possibilities of democratic revitalization within the historical trajectory of capitalist liberal democracy. There is, however, a kind of civil society engagement that radically steps outside of the colonizing paradigm of the system. Prefigurative politics, defined as “the embodiment, within the ongoing political practice of a movement, of those forms of social relations, decision-making, culture, and human experience that are the ultimate goal” (Boggs, 1977; p. 100), assumes that desired social change will not happen within the constraints of the existing social-political order: thus, neither working within that order nor criticizing it suffice. Marxism, anarchism, Gandhism, and indigenous politics all have prefigurative traditions, in which a community turns away from existing socio-political organization and seeks to establish social forms and norms that respond to the perceived shortcomings of the mainstream organization.

It would be naive to suppose that most prefigurative projects can entirely insulate themselves from the dynamics of the system. However, the aspiration to do so is itself powerful: prefigurative politics can identify and critique premises of prevalent socio-political forms that we may be blind to when working within them. Prefiguration can de-normalize the prevalent socio-political organization and thus highlight the contingency of features that, within their own paradigm, appear natural. This is particularly useful with respect to capitalist liberal democracy, which has historically presented itself as a transcultural, civilizational project. And, of course, prefiguration goes beyond critique for its own sake: it attempts to generate alternate dynamics, and to provide a distinctive social setting in which those dynamics can flourish. In effect, prefigurative practice aspires to show us the beginnings of a future different from the ones we might imagine emerging from the prevalent socio-political order.

For the purposes of Goal 16, then, rather than (solely) focusing on re-democratizing the prevalent socio-political order, it would be helpful to identify a prefigurative community attempting to build a new model—one where, rather than have the system be in a relationship of colonization with the lifeworld through bureaucratic power, system and lifeworld mutually cultivate communicative action in each other. By identifying the premises of the prevailing system that such a prefigurative practice critiques, and the alternative premises that it seeks to build upon, we may draw lessons for mainstream political institutions.

## 5. The Bahá'í community as a prefigurative resource

The global Bahá'í community presents an intriguing example of prefigurative practice. The Bahá'í Faith is a global religious community, distinguished by extreme ethnic and linguistic diversity despite its relatively small size (Smith P., 2022)^2^, as well as by a democratic institutional structure that is conceived of as a pattern for future developments in the wider world. In its internal democratic functioning, the Bahá'í community can be thought of as prefigurative: it seeks to embody “the ultimate goal,” and conceives of itself as building something new.^3^

The Bahá'í example is particularly relevant to the question of how to advance Goal 16 in light of Habermas' insights into the importance of, and modernity's deficits in, communicative action. The Bahá'í community self-consciously engages with the question of how to build a social order infused with communicative action, both in the lifeworld and in the more formal institutional context where Habermasian analysis would expect system-like coordination to prevail. Bahá'í practice along these lines is informed by social theory simultaneously rooted in religious texts and framed as a body of knowledge that evolves through experience.

I will first consider the *telic-*organicist Bahá'í conceptualization of institutions in relation to community and individual.^4^ The conception is organic in that it theorizes unity in diversity as the ideal that should inform practice, and *telic* in positing a purpose and goal for both the human being and society as a whole. In both respects, the model, while retaining a place for specialized governance institutions, softens the boundary between lifeworld and system; it also rejects the individualism underlying the prevalent socio-political order.

Second, I will consider the practical means by which the Bahá'í community attempts to fulfill its explicit commitment to fostering and protecting genuine communicative action as the primary means of organizing the relationships between individuals, communities and institutions, in line with the *telic-*organicist model, and suggest that this combination of ideals and practice can reasonably be hoped to resist the colonizing relationship between system and lifeworld.

### 5.1. Reconceptualizing system and lifeworld as an organic whole

Bahá'í prefigurative practice implicitly critiques both of the mutually reinforcing elements identified above as contributing to the disintegration of the public sphere, viz. the individualistic conception of human nature, and the colonizing relationship of system and lifeworld. The former critique is not dissimilar to that found in certain other prefigurative traditions. Gandhi's approach to social change, for example, centers on a cultural shift in which the power of the human desire for truth, channeled through religious and cultural resources present in the lifeworld, would act to reinvigorate that lifeworld with new possibilities in the form of interrelationships based on altruistic service rather than competitive individualism. However, a Bahá'í critique of the structural position of institutions—particularly governance institutions—in the modern world is distinct. Unlike prefigurative Gandhism or some versions of Marxism, the Bahá'í framework takes institutions as an integral part of its model for human society. Gandhi believes that were the people to attain spiritual mastery of selfish impulses, the state would have no further role, as justice would be built through the relationships between individuals expressed in communities (Parekh, 2001). In contrast, a Bahá'í framework does not relegate the state to an instrumental and temporary role in this way; nor does it take for granted the opposition of lifeworld and system. Instead, it models a set of organic interrelationships between individuals, institutions, and community. The Bahá'í model agrees with Habermas (contra Gandhi) that the coordinating function of the system cannot simply be removed from modern societies without a catastrophic collapse. It thus seeks to grow its own institutional forms as part of its prefigurative practice; this growth occurs within the matrix of existing societies, rather than in communities deliberately (geographically) removed from wider society like Gandhi's ashrams.

#### 5.1.1. The human body metaphor

The Bahá'í conception of the oneness of humanity helps illuminate the nature of its organic model for social organization. The Faith's founder, Bahá'u'lláh (1817–1892), wrote that, in spite of its failure to realize this truth historically, humanity is ontologically one (Bahá'u'lláh, 2020a; *Tabernacle* 1.15), and that this oneness would begin to be realized consciously, and incorporated into the global social order (*World Order* 28 November 1931). This unity is multifaceted: beyond merely a condition of outward peace between nations and individuals, it entails an attitude of oneness, rooted in individuals' hearts and minds, and permeating all social arrangements and institutions (Effendi, 2023). An idea of oneness—of the divine, of creation, and (explicitly or implicitly) of humanity—is of course common in religious thought. What is distinct about the Bahá'í concept of human oneness is not only its centrality as a doctrine (see next section), but that it explicitly influences the details of social and political organization—i.e., of the life of the community, the role of institutions, and the way in which individuals interact with both as well as each other—not only as they are worked out within the community itself, but as they are enshrined in the core, authoritative religious texts of the Faith. These details, and their connection to oneness, will be explored in subsequent sections of this paper, but it is worth first elaborating on the conceptual spiritual-ethical framework that informs these specifics.

In many of their undertakings, Bahá'í communities—both at the global and the local level—return to a metaphor found in the scripture of their Faith: humanity as a human body. This metaphor finds a number of expressions in Bahá'u'lláh's writings. Specifically, it is often used to highlight that the suffering of any part of humanity is the suffering of all: “the whole human race is encompassed with great, with incalculable afflictions. We see it languishing on its bed of sickness, sore-tried and disillusioned” (*Tabernacle* 1.5; Bahá'u'lláh, 2020a). At their core, these “afflictions” are not externally imposed harms—no part of humanity is an “other” responsible for harm to “us” —but manifestations of an internal disordering of the body itself:

Regard ye the world as a man's body, which is afflicted with divers ailments, and the recovery of which dependeth upon the harmonizing of all of its component elements. (*Summons* 152; Bahá'u'lláh, 1952)

This organic metaphor has, of course, been explored in other contexts, and put to a variety of uses. Bahá'u'lláh's immediate audience in the Persian cultural sphere would identify it with the poet Saadi, who in his famous poem “Bani Adam” likens all human beings to parts of a shared body, stressing the resulting claim that each has on all. Conversely, Herbert Spencer uses the human body analogy to illustrate the functionalist view of society as an organic entity, likening institutions to the body's organs that work together to ensure its proper functioning.^5^ The image can also be used to express hierarchical ideas of social organization, as in the medieval concept of the “body politic” whose “head” is the monarch, or Hobbes' famous image of the sovereign as a Leviathan, a composite of all the persons in the realm who bestow their power and natural freedom upon it.

A metaphor, of course, invites interpretation. There is a world of difference between how Saadi, Spencer, and Hobbes use the human body image. In the Bahá'í tradition, the metaphor is not restricted to explaining the relationships between individuals, nor those between institutions; neither is it used to privilege the collective over the individual, or to justify a social hierarchy. Instead, interpreted through the lens of the particular worldview that Bahá'u'lláh presents in his teachings, the metaphor accentuates the dynamic interplay between society and its component parts—individuals, institutions, and communities—emphasizing that each contributes in a dynamic and progressive process to the others' thriving (Smith T., 2020).

#### 5.1.2. Unity in diversity and institutional implications

This understanding of the metaphor begins with the premise that humanity is, fundamentally and ontologically, one, and is healthy to the extent that its common spiritual origin is reflected in a harmonious state of existence on the planet that it shares. This idea of the oneness of humanity, described in authoritative Bahá'í texts as “the pivot round which all the teachings of Bahá'u'lláh revolve” (*World Order* 28 November 1931; Effendi, 2023), is often cited by Bahá'ís as the religion's core teaching. Thus, Bahá'u'lláh writes:

Know ye not why We created you all from the same dust? That no one should exalt himself over the other. […] Since We have created you all from one same substance it is incumbent on you to be even as one soul […] that from your inmost being, by your deeds and actions, the signs of oneness and the essence of detachment may be made manifest. (*Hidden Words* Arabic 68; Bahá'u'lláh, 2020b)

The appeal to the individual to “know” their oneness with the rest of humanity reinforces the individual/spiritual foundation of the social harmony being called for. But the body metaphor goes beyond a call to spiritual unity; it also accentuates differentiation. That humanity is in need of “the harmonizing of all of its component elements” highlights the irreducibility of difference in its parts: a state of harmony exists between things that are distinct, like notes unified in a chord. Thus, the unity the Bahá'í community pursues is unity in diversity. The Bahá'í International Community, the NGO that represents the global Bahá'í community in international fora, puts it this way:

[…] Bahá'u'lláh compared the world to the human body. […] Human society is composed not of a mass of merely differentiated cells but of associations of individuals, each one of whom is endowed with intelligence and will; nevertheless, the modes of operation that characterize man's biological nature illustrate fundamental principles of existence. Chief among these is that of unity in diversity. Paradoxically, it is precisely the wholeness and complexity of the order constituting the human body—and the perfect integration into it of the body's cells—that permit the full realization of the distinctive capacities inherent in each of these component elements. No cell lives apart from the body, whether in contributing to its functioning or in deriving its share from the well-being of the whole. The physical well-being thus achieved finds its purpose in making possible the expression of human consciousness; that is to say, the purpose of biological development transcends the mere existence of the body and its parts (Bahá'í International Community, 1995).

Rights and duties are thus inextricable: every cell has a claim on the body, and vice versa. But precisely because the cells are differentiated, there is also a need for coordinating entities, such as organs, within the body as a whole. Crucially, the organ has no meaning outside of the body: it is sustained by the body and exists to serve the entire body. If it takes any model for its operation other than service to the whole, the result is disastrous—consider autoimmune disease, where the immune system attacks another component of the body.

It follows from this organic model that, in the Bahá'í understanding, institutions do not simply fulfill the negative, Madisonian role of safeguarding society from the consequences of human imperfections—a role that would theoretically diminish as the community's vision for human spiritual perfection is progressively attained. Instead, the institution has a positive role; it makes contributions to the common good that individuals and communities, however advanced, cannot make.

#### 5.1.3. A telic understanding of individual and society

This points to another dimension of the Bahá'í paradigm: historical consciousness (Smith T., 2020; Berger, 2021). As is common in religious traditions, the Bahá'í model conceives of the human being as an entity with a purpose, a *telos*. This purpose is framed in a variety of complementary ways, but can be summarized as the progressive development of latent spiritual qualities (analogous to virtues in the Aristotelean tradition), accomplished principally through service to other human beings. However, somewhat distinctly from most religious traditions—and in a way that resonates more with modernist thought, such as Hegel's—human history is also *telic*, because human beings “have been created to carry forward an ever-advancing civilization” (*Gleanings* CIX; Bahá'u'lláh, 1949). Humanity's goal is not to reach a state of static perfection, but a condition of progressive organic growth, betterment, and refinement at both the individual and collective levels. This historical process does occur as a mere product of impersonal historical forces; it is at least partially determined by human choice, as individuals and communities struggle to determine how to adapt to changing conditions. Individuals' efforts to carry out their own *telic* purpose—virtue development through service to humanity—are integral to carrying forward an ever-advancing civilization. Civilizational advancement also necessitates an element of coordination that requires institutional involvement, as illuminated by the human body metaphor.

Crucially, Bahá'ís believe that our time is of particular significance in this historical process: “[h]umanity, it is the firm conviction of every follower of Bahá'u'lláh, is approaching today the crowning stage in a millennia-long process which has brought it from its collective infancy to the threshold of maturity—a stage that will witness the unification of the human race” (Universal House of Justice, 2013). Faith that this unification—the state of harmony described above—is attainable, and conviction that its attainment requires concerted human effort, means the *telos* of the human being interacts with the distinctive *telos* posited for human history to produce a kind of political agency in which individuals, communities, and institutions aim, in all political undertakings, to both achieve outcomes that enhance social harmony (typically referred to as “unity” in the Bahá'í context), and to adhere to processes consistent with such harmony. In its role as a central spiritual quality in the *telic* progress of the individual, unity precludes conflict as a methodology; where differences between views arise, then, they must be dealt with in a way that fosters unity. This attempt to embody in practice the very culture, relationships, etc. that are the ultimate goal is, as noted above, a hallmark of prefiguration.

This ethos of unity informs not only relationships between individuals, but those between individuals, their communities, and institutions. When the imperative of personal spiritual growth through service is combined with the metaphor of the human body and with the developmental view of history, a particular kind of mutualistic relationship between individuals, their communities, and institutions becomes conceivable. This relationship is characterized by reciprocal improvement through mutual capacity building. The individual and community see their institutions as nascent, organically growing, and in need of active engagement with the community and individuals in order to increase their capacity. In this vision, while the distinction between lifeworld and system remains meaningful for some purposes (i.e., the “state” does not disappear), the radical tension between them is defused. The system does not pursue goals distinct from the well-being of the whole and each of its parts.

We can briefly consider how the view of history outlined here permits a different perspective on the problem of communicative action given the modern relationship between system and lifeworld. Where Habermas uses structural analysis to discover how the historically recent phenomenon of communicative action in the political realm became fettered, the *telic* view of history takes modernity not as an endpoint to be explained, but a moment of transition between humanity's childhood and its maturity. Humanity has, in this adolescent period, seen new powers awaken, that of communicative action not least among them—but it has not yet arrived at a mature framework within which to exercise this power. The challenge set before the Bahá'í community, and which it sees as confronting humanity as a whole, is to develop such a framework, based on a foundation of core ideas, norms and practices, but informed by learning through experimentation.

What has been described so far falls within the realm of ideal theory. Far from naively asserting that the above vision will suffice to avoid the colonizing dynamic Habermas highlights, the Bahá'í community has, since its inception, set about deliberately accumulating experience with a set of norms and practices intended to give the vision concrete reality. These norms and practices, insulated to some degree as prefigurative practice from the socio-political mainstream, can be viewed as a construct designed to nurture and protect communicative action as the means of generating collective will and channeling collective power.

### 5.2. Fostering communicative action: norms, practices, and structures

While a full description of a Bahá'í approach to governance is not possible here, two important facets help illustrate how the organic conception of institutions, individuals and communities, and the *telic* conception of individual and of history, play out in ways that may suggest under what conditions a public sphere, and its relationships to institutions, might sustain communicative action as the primary mode of coordinating society. The first facet is that of *consultation*, while the second is that of the *Administrative Order*, the formal structure through which Bahá'ís govern their affairs.

#### 5.2.1. Consultation as communicative action

A Bahá'í model seeks to ensure that the right conditions are established and maintained for communicative action to flourish. Central to this is “consultation,” a Bahá'í practice that fulfills an equivalent function to rational-critical debate, although it rests on certain ethical/spiritual foundations that somewhat nuance this term. I therefore suggest that the Bahá'í model espouses *rational-critical consultation* as the ideal of communicative action. As will be seen, consultation challenges the individualist premise of liberal democracy, and in its use of consultation, the Bahá'í community seeks to prefigure a different kind of culture that can nevertheless be accurately described as democratic in both a vote-centric and talk-centric sense.

Bahá'í consultation denotes non-partisan discussion prioritizing group unity and the collective search for truth over adherence to ideological or personal positions. “Consultation, frank and unfettered” is prescribed as “the bedrock of this unique [Bahá'í Administrative] Order” (*Consultation* 27; Bahá'u'lláh et al., 2023). However, its applicability is not limited to formal institutional settings: “[i]n all things it is necessary to consult” (*Consultation* 5; Bahá'u'lláh et al., 2023), whether within a family or amongst the entire community, and whether the goal be to reach a decision, bring new insight to a problem, define a range of possibilities for action, make a recommendation to a formal Bahá'í institution, achieve greater unity of thought, or simply learn together and develop collective understanding. Karlberg, who frames consultation's general purpose as the “collective investigation of reality… in a manner that promotes unity and justice,” highlights three functions of consultation:

In some contexts, it is *exploratory* in nature, with the purpose of generating collective awareness, insight, and understanding regarding an issue of common interest or concern. In other contexts, it is *advisory* in nature, with the purpose of providing advice, feedback, suggestions, or constructive criticism to those who will be making decisions. And in other contexts, it is *decisional* in nature […]. All these functions can be expressed in formal and informal ways, through communicative processes playing out in an ongoing manner at all levels of community life (Karlberg, 2018; p. 81).

Bahá'ís thus view consultation as a component of their community culture from the level of the family upwards. In a very real sense, the enormous emphasis placed on consultation as a *modus operandi* for investigating truth, making decisions, etc. means that the Bahá'í community espouses a normative commitment, reinforced by practice in community spaces, to communicative action as an essential instrument for generating collective will and applying collective power aimed at cultivating individual and social betterment (Smith and Ghaemmaghami, 2022).

A few salient features or norms of consultation can be noted, adherence to which allows Bahá'ís to recognize a process as consultative. Participants are to express themselves with courtesy, and complete honesty. Differences of opinion are prized, as the “shining spark of truth cometh forth only after the clash of differing opinions” (‘Abdu'l-Bahá, 2023, p. 44). A view, once stated, becomes the property of the group; in the interests of truth-seeking, participants should endeavor to be detached from their own views (Smith and Ghaemmaghami, 2022). When the goal of consultation is to reach a decision, consensus is ideal, but a majority decision is acceptable. Crucially, there is no concept of “dissent” in consultation: every participant—and, in the case of a decision by an Assembly, all members of the community—are encouraged to lend their support to the decision, regardless of whether they agree with it (‘Abdu'l-Bahá, 2023, p. 45). Unity behind a decision is of greater importance than the correctness of the decision. This principle rests not only on the importance of unity for its own sake as a paramount good, but also on the belief that so long as the group remains united, it can learn together, and later unitedly correct a decision that proves wrong; whereas a practice of not unitedly carrying out decisions will prevent correct decisions from taking proper effect (*Consultation* 12, 15; Bahá'u'lláh et al., 2023).

These features of consultation are coherent with the Bahá'í concept of human nature described earlier, in which the human's purpose is to acquire spiritual virtues through service to humanity. Such a human is rational, and their legitimate self-interest is not denied, but because it is also not *centered*, it is possible to pair honest expression of one's views with a willingness to detach from them, change them, and/or subordinate them to the group decision. In a Bahá'í conception of individual *telic* progress, success in advancing one's own views for their own sake is not prized. Conversely, attaining greater understanding is highly prized, and here consultation plays an important role: “[t]he maturity of the gift of understanding is made manifest through consultation” (*Consultation* 3; Bahá'u'lláh et al., 2023). Since consultation is itself premised upon the ethical orientation of participants (devotion to truth, detachment from personal views, welcoming contributions from all, etc.), growth in understanding and in virtue are inextricably bound, and collective, communicative action is vital to both. The path to individual betterment in this model is thus fundamentally interrelational and communicative.

Bahá'ís, of course, do not use the phrase “communicative action” in describing consultation. To support the claim that consultation qualifies as communicative action it is worth briefly considering two frameworks Habermas provides for assessing the quality of communication. The first, that of the ideal speech situation, describes criteria under which participants in communication are able to evaluate each other's claims based solely on reason, free of overt or socially-embedded coercion. These criteria are that every person who is competent to speak is permitted to do so, and can both present their own views and assertions and question the assertions of others (Habermas, 1990). These criteria are broadly met by consultation. While it might be argued that the normative emphasis of consultation on sincerity and courtesy exerts a psychologically coercive influence on participants, leaving them less than totally free to voice any possible assertion, this may not be a deficiency if considered through the second framework, that of the validity claims implicit in communicative action. Habermas outlines three validity claims that are typically assumed, and unremarked, in our speech, but that must be available for interrogation when disagreement occurs in a discussion. A speaker is assumed to make the implicit claim that their speech meets the criteria of truth (in the sense of accurate reflection of objective reality), normative correctness, and sincerity, and is thus valid.^6^ Consultation, viewed as a cultural practice, places a heavy emphasis on sincerity, as noted, and seeks to elicit both forthrightness and detachment. From this basis, its main focus is precisely on the assessment of truth and normative correctness, the former by collective examination of available evidence, and the latter using the standard provided by the collective understanding of relevant spiritual and social norms. While consultative participants are normally assumed to be sincere, each is encouraged to *not* assume the absolute truth of their own words: truth is held as an open question for the group to assess. Some statements, of course—descriptions of one's own feelings for instance—the speaker is unlikely to be persuaded to be false; but even these can shift and be nuanced by the deliberations of the group. In its focus on the collective search for truth and normative correctness, consultation resonates with Habermas' description of the rational discourses central to both science (which takes the assessment of grounds of truth as its focus) and law/ethics (which focuses on assessing arguments about normative correctness).

While Bahá'ís would not assert that, individually or collectively, they have learned to consult perfectly, their efforts to develop their capacity to consult have contributed to the unity of their global community across local contexts as culturally diverse as can be imagined. Further, faith that genuine consultation *can* be achieved, based on a concomitant faith in human nature as potentially altruistic and capable of progressive spiritual growth, motivates the attempt despite the challenges it poses and the contrast it presents to the political norms of wider society.

#### 5.2.2. Formal structures: institutional rules and norms

Having no clergy, the Bahá'í community conducts its affairs by means of an internal administration which Bahá'ís understand to reflect a divine plan. This “Administrative Order” has its origin in the teachings of Bahá'u'lláh, and features democratic institutions, organically unified across the entire planet, and operating deliberatively within themselves and in relation to the community.^7^ It is also, in all its facets, explicitly non-adversarial.

At a formal level, the Administrative Order includes local, national, and in some places regional elected bodies (“Assemblies”), as well as a global body, the Universal House of Justice. While mapping concepts developed in the context of secular politics onto a religious community can be somewhat misleading, the Administrative Order broadly combines judicial, executive, and in the case of the Universal House of Justice, legislative authority in the same institutions.

All Bahá'í elections are devoid of nominations, campaigning, and parties. These are formally prohibited by rules, reinforced by strong norms—in other words, an individual or group's perceived attempts to campaign or form a faction will tend to make Bahá'ís less likely to vote for them (Abizadeh, 2005).

The prefigurative lens illuminates the rationale behind these rules and norms. They are not based on a judgment that adversarial politics has no social utility; partisan political processes can lead to good results, not least the day-to-day maintenance of polities that (by historical standards) are remarkably safe and prosperous. It is instead based on an evaluation that the premises, practices, and consequences of partisanship, whose methodology is adversarial, are not fully compatible with building the new socio-political structure that is to accommodate humanity's further progress in the future. Nor, from the standpoint of the human being's *telos*, is division of people into oppositional camps compatible with the individual's growth in the virtue of oneness. Political parties, campaigning, etc. may still be necessary for maintaining the existing socio-political structure; they are simply not avenues of social contribution open to Bahá'ís, who are encouraged to focus on learning what a new structure may look like.

Since the means to actively seek any position of leadership within the community are removed, politics is not a profession in the Bahá'í community (Universal House of Justice, 1996). Thus, any adult member of the local community can both vote and be voted for. Each voter lists (by secret ballot) the nine individuals they feel best qualified for membership on the Assembly, and the nine who receive the most votes are elected. Nomination is eschewed on the principle that it limits both the freedom of the elector and their responsibility to become sufficiently involved in their community to choose for themselves who to vote for. There is a strong norm against discussing individuals' suitability for election, although discussion of the general qualifications for election is encouraged (Abizadeh, 2008). The system is thus designed to both prevent power-seeking and make it counter-normative. Furthering this reconceptualization of authority, no elected individual has any personal authority whatsoever in the community by virtue of their election; thus, an Assembly can only make decisions as a body [Bahá'í Administration 11 April 1933; Effendi Bahá'í Administration, (n.d.)]. The institution thus has a completely distinct identity from the individuals currently elected to it.

This model scales through a series of representative elections. Thus, in addition to electing their own local Assembly each year, Bahá'ís in a locality will elect delegates (using the same procedure) to a yearly National Convention. The delegates at the Convention will both consult on the issues confronting the national community and elect a National Assembly (voting for any adult Bahá'í in the country is permissible). Finally, the combined membership of all National Assemblies meets every five years to elect the Universal House of Justice.^8^

While Bahá'ís would generally accept that their understanding of, and adherence to, the standards set out in their authoritative writings for this election process are still maturing, the basic rules and norms described above are observed with a remarkable degree of fidelity. So too is the norm that the results of an election are to be “conscientiously and unquestionably accepted by the entire body of the believers, not necessarily because they represent the voice of truth […] but for the supreme purpose of maintaining unity and harmony in the Community…” (Effendi and the Universal House of Justice, 2023, p. 16). Bahá'ís are encouraged to view their local and national assemblies with an attitude of respect and love, and be forbearing of errors and growing pains.

This model of representative democracy acts to promote and protect communicative action, not only within the “public sphere” (read as the Bahá'í community), but as the primary means of interaction between individuals, community, and institutions. The elected Assembly is charged with maintaining awareness of the views and needs of the community, which are brought to the fore through consultation in recurring community gatherings and spaces (including the Nineteen Day Feast, held once a Bahá'í month, and the Reflection Gathering, held once every three months in many localities). The Assembly is also responsible for conducting ad hoc consultations with individuals and groups as necessary. However, while its own decision making occurs through internal consultation, members, and the Assembly as a whole, are *not* tasked with representing the will of either the people who voted for them^9^ or the community as a whole. Instead, their responsibility is to their own conscience, meaning their understanding of how the teachings of the Bahá'í Faith apply given whatever circumstances are under consideration, with a view to the best interests of the whole community (not limited to the Bahá'í community). Governance is thus not a matter of negotiating irreducible interests; the processes of public will formation within the community, and of Assembly decision making, are both conducted according to consultation. By the same token, the Assembly and its members, while they communicate relevant decisions to the community, cannot seek public “acclamation” for them, because the members cannot campaign for re-election. The lack of campaigning, nominations, partisan apparatus, etc., means that the “publicity” model of politics that emerged within liberal democracies in the age of mass communication cannot easily arise. There is no legitimate mechanism within this system by which an Assembly, or a member thereof, can attempt to mobilize public opinion in a certain direction.

The norms and rules structuring the relationship between the “system” and lifeworld in a Bahá'í community warrant consideration as a potential means to cultivate and preserve communicative action as the mode of coordination. These norms and rules are not arbitrary, nor are they conceived of primarily as responses to perceived shortcomings of liberal democracy, or any other specific system of human governance. Instead, they are coherent outgrowths of a positive vision of human nature and society, consisting of a range of foundational premises. While these premises are too numerous to review in this paper, the two that I have highlighted are (a) the oneness of humanity, understood as implying the possibility of organic harmony between individuals, communities, and institutions, and (b) the *telic* understanding of both the human individual, as capable of developing spiritual (altruistic) qualities, and of humanity as a whole. By connecting communicative action-enhancing norms and rules robustly to a coherent vision for human nature and society, a Bahá'í political framework invites consideration as a prefigurative model of how system and lifeworld can avoid the colonizing relationship whose historical origins in liberal democracy Habermas traces in *Structural Transformation*.

#### 5.2.3. A culture of learning: the self-reflective nature of Bahá'í prefiguration

The dynamics of communicative action in a Bahá'í model are not applied only to the quotidian issues that arise in the community. In keeping with the *telic* model of history, the community conceives of itself as a body in a process of organic growth and development. This development occurs as individuals, communities and institutions engage in mutual capacity building, as noted above; communicative action is at the heart of this process. The emphasis on consultation in all community spaces casts individuals as protagonists in the generation of knowledge—roughly, information (data) gleaned from experience, mapped onto concepts that are either generated spontaneously or are already current in the community. Community spaces such as the monthly Nineteen-Day Feast and periodic reflection gatherings allow this consultatively generated knowledge to be shared with local institutions, which in turn relay knowledge up to national, and through them global, institutions, while the yearly national convention allows learning to be shared and disseminated directly from local communities to the national unit. Simultaneously, knowledge is disseminated “downward” through this system: having been systematized and generalized at a national or global level, learning is shared with the community—in effect, the community is shown what it has collectively learned—through regular communications.

This downward dissemination of knowledge also instills communicative action into the exercise of authority. Institutions do not generally act by fiat on matters of general concern to the community, but instead guide the community through letters—rational-spiritual texts that are studied in group settings, and become the basis for further consultation.

A recurring theme in letters from the Universal House of Justice is that the Bahá'í community is encouraged to conceive of itself as engaged in a process of learning about a new social-political order, through experimentation within the guiding constraints of prescribed institutional forms, norms, etc. (Karlberg and Smith, 2022; p. 466). This orientation— referred to as a “culture” or “mode of learning” by the Universal House of Justice (2010)— results in a political culture that stands in contrast to mainstream liberal democracy. In both, the goal of collective (political) activity can be conceived of as development, broadly speaking. However, whereas in liberal democracy, the vision for, and authority over, development is negotiated and contested through political conflict (between parties, etc.), in the Bahá'í context development is an object of collective learning, advanced through the mutualistic process of capacity building. This understanding is reflected in the following passage from a letter by the Universal House of Justice. While written in the context of socio-economic development, it reflects concepts and learning honed within the socio-political context of the Bahá'í community's own growth and development:

When development is seen in terms of the participation of more and more people in a collective process of learning, then the concept of capacity building assumes particular importance. […] Setting and achieving specific goals to improve conditions is a legitimate concern of social action; yet, far more essential is the accompanying rise in the capacity of the participants in an endeavor to contribute to progress. Of course, the imperative to build capacity is not only relevant to the individual, important though that may be; it is equally applicable to institutions and the community, the other two protagonists in the advancement of civilization (Universal House of Justice, 2012).

## 6. Discussion

Having outlined the case for Bahá'í prefigurative practice as a relevant model for thinking about how to overcome the colonization of lifeworld by system posited by Habermas, and to infuse relationships between individuals, communities and institutions with communicative action as a mode of social organization, it is now possible to extract insights into how Goal 16 might be achieved. I will first present possible strengths of the Bahá'í example with respect to institutional effectiveness, inclusivity and accountability, before considering how, practically speaking, similar features might be fostered in mainstream governance institutions. This will include consideration of what it means to look to a religious community to illuminate socio-political questions.

### 6.1. Strengths of the Bahá'í prefigurative example with respect to Goal 16

The form of universal democratic participation in the Bahá'í model, to the extent that it centers the communicative action that in Habermas' analysis has eroded in the wider public sphere, may suggest a path toward greater institutional inclusivity and accountability without undermining institutional effectiveness.

First, inclusivity of individuals in institutional processes within this model is potentially bolstered by a robust sense of ownership. Because voting is inextricably linked with consultation—in the immediate context of an election, and more generally throughout community life—elected institutions of the community can be genuinely perceived as an expression of the community itself. The preclusion of parties and campaigning prevent factionalism from intervening between the community and its institutions, which thus avoid being perceived as representing only some. Individuals can thus begin to see their institutions as the collective project of their community, a project they are actively nurturing and helping to develop. The institution understands itself in these terms as well: institutional actors adopt humility and a posture of learning in their official capacities. The institution also takes responsibility for nurturing its citizens' capacities—through elicitative communicative action, not coercion—which constitutes another dimension of inclusivity.

Second, the focus on knowledge as a shared resource, whose richness requires universal participation and a diversity of perspectives at the level of inputs, consolidation by institutions (ultimately at a global level), and equal dissemination to all individuals and communities, enhances both effectiveness and inclusivity.

Third, the non-adversarial dynamics of the formal electoral process foster inclusivity, as well as accountability of a certain kind. Specifically, while the decisions of an elected body are not subject to the approval of the electorate, this electorate is to consider the qualifications required of those who are to be elected, and vote in such a way as to ensure that the individuals selected, and the overall composition of the body, reflect these qualifications (Abizadeh, 2008). The centrality of consultation also enriches the concept of accountability. While institutions have a specialized role, all community members are in some measure accountable to each other, themselves, and the community as a “body politic” in terms of fulsome participation in consultation and adherence to its norms. This emphasis on process, which is within the power of each to strengthen, is arguably more empowering than the focus on outcomes which prevails in Habermas' transformed public sphere. There, with democratic participation for the masses largely reduced to acclamation, the individual voter can easily feel as though they ultimately have no real impact on their political community.

Finally, the global integration of the Bahá'í model is worth noting. Driven by the global vision embedded in the structure of Bahá'í administration from its inception, the model unifies culturally diverse communities across the globe, leading to dynamic possibilities in terms of sharing, disseminating, and localizing knowledge and resources. Since cooperation is the premise and default arrangement, a truly global mobilization behind development, in response to issues both urgent and mundane, is not only possible, but is the norm.

Put differently: in principle, at least, the Bahá'í model achieves deliberative democracy at scale. Within a small geographic context—a neighborhood or village—universal participation in consultation is feasible. At higher levels of organization, the electoral system—in which the means of power-seeking are as much as possible stripped away by both rules and norms, and in which consultation with the electorate features as part of the process—allows deliberation to permeate institutions and their relationship with communities, answering the critical challenge of how deliberative democracy can operate meaningfully at scale.

### 6.2. How can the prefigurative example serve?

The question that remains is how a prefigurative example like the Bahá'í model can practically serve to inform institutional progress in the wider world. As in many prefigurative traditions, there is an idea within the community that what is being built will one day play a role in re-shaping human socio-political organization. Indeed, like liberalism itself, the Bahá'í Faith is explicitly a civilizational project that aspires to universality: it not only hopes, but confidently predicts, that it will in some way contribute to the creation of an unprecedented global socio-political order.

However, where some prefigurative traditions might hold that they have nothing to say to existing socio-political structures, which they anticipate will eventually be replaced, the Bahá'í conception of its own contribution in this regard is, like its internal organization, informed by a vision of harmonious cooperation. Thus, coherent with features of the Bahá'í framework including consultation and a culture of learning, the Bahá'í community explicitly invites examination of its experience, and aspires to contribute to constructive discourses aimed at social progress. This is possible in part because while—like all prefigurative practice—its critique of political modernity (including liberalism) is so fundamental as to make it necessary to turn away and create something new, it nevertheless does not *reject* liberalism; instead, it contextualizes it within a *telic* understanding of human history, allowing it to acknowledge liberalism's advances, and invite both its defenders and critics to reflect on possibilities for improvement.

If a Bahá'í model is to have any influence on socio-political arrangements, it will generally be through the lifeworld that it shares with wider society. As a prefigurative tradition—and in keeping with its normative commitment to non-power seeking within existing political systems—the Bahá'í community does not attempt to impose its concepts or practices on anyone. The community is instead explicitly committed to a form of community building in all social contexts, in which distinctions between religious adherents and others are minimized (Karlberg and Smith, 2022). As such, it aspires to bring its dynamics of communicative action into the lifeworld. To the extent that these practices, and the ideas they rest upon, can take deeper root in the lifeworld, they will provide an ideational and experiential resource, available to be taken up into the existing system. This might occur by way of discourse in the public sphere; additionally, lifeworld contexts outside the public sphere, including individuals' moral and intellectual formation within families, friend groups, etc., may directly interact with the system. Ultimately, the actors who pull the levers of the system are themselves part of the lifeworld; to the extent that that lifeworld can not only resist colonization, but establish new bases of communicative action, it can come to inform their actions in the system.

### 6.3. Religious communities as resources for institutional change

It must be recognized that in the Bahá'í example, faith has historically acted as a vital catalyst for the gradual development of the framework described here. This is more than faith in the possibility of a better socio-political organization; it is, at its core, religious faith that the framework the community is trying to build is divinely designed and supported. It may nevertheless be that, after a hundred years of experience in building its Administrative Order, and more than that in practicing its consultative methodology—not only internally, but in broader community-building work in which the distinction between Bahá'ís and others is increasingly de-emphasized (Karlberg and Smith, 2022)—the Bahá'í community's body of experience can satisfy the observer on its own merits.

To consider how the ideational influence in the lifeworld described above might occur, we can consider how the contributions of religious communities to political discourse in liberal societies have been theorized. This is a question that Habermas in particular has given greater attention to since *Structural Transformation*.

Habermas takes seriously the capacity of religious communities to “become a transformative force in the center of a democratic civil society,” particularly when their distance from secular positions “on normative issues… stimulate[s] an awareness of their relevance” (Butler et al., 2011; p. 25). Habermas' gloss on the Rawlsian concept of public reason can be taken as a paradigm for thinking through a Bahá'í model's possible utility in the public sphere. Habermas suggests that a religious community's contributions to public discourse in civil society can, and should, be on its own terms: no additional burden of conveying religious ideas in terms accessible to all, regardless of their comprehensive doctrine, should be laid on religious citizens or groups as a price for their engagement in the public sphere. Only the “formal deliberations of political bodies that yield to collectively binding decisions” must be in such universal terms; here, religious contributions must be “translated” accordingly (Butler et al., 2011, p. 26).

Thus, Habermas would suggest that insights from a Bahá'í model can be presented on their own terms within conversations in the public sphere in a pluralistic society. It may nonetheless be instructive to engage in a kind of translation, if the goal is not merely to present the example, but to present it in a way that makes it intelligible to those of a variety of backgrounds. This is something the Universal House of Justice invites the Bahá'í community, its institutions and the individuals within it, to learn about doing, by “contributing to the discourses of society” (see for instance Universal House of Justice., 2021).

I will therefore conclude this article with a preliminary sketch of what such translation might look like.

I have suggested above that a Bahá'í model of democratic governance operating via communicative action can be reconstructed on the basis of a few core premises. These premises, then, are what would need to be translated in order to achieve a sufficient basis of acceptance to warrant wider adoption of (elements of) the model.

The three premises that I have focused on here are the oneness of humanity, the *telic* narrative of human history, and the *telos* of the individual human being. The first two do not strike me as too challenging to translate. A concept of global human solidarity, if not the dominant strain in our global culture, is a clear and increasingly powerful one. The idea of a direction to human society is also not foreign to mainstream political culture; in liberal democracies, the narrative of social movement toward a perfection of human freedom is current, for instance. A Bahá'í model derives particular implications from both principles, however, based on their interaction with the third, the *telos* of the human being as development of spiritual virtues. Through this lens, for instance, the oneness of humanity must be advanced through means that help develop, or at least do not hinder, the individual's spiritual growth. This in turn precludes political practices that treat anyone as an other. Similarly, if the *telos* of humanity as a whole is understood as advancement toward harmonious unity in diversity, then not only must the means used to advance toward this be commensurate with the goal—a common feature of all prefiguration—but they must not impair the individual's development of unity, in thought and action, with others.

How then can the *telic* story of the human being be translated into broadly intelligible terms? This may not be so difficult as it seems. Viewed in the global context of religious, spiritual and ethical teachings, it is not difficult to find resonance with this view of the human being. Indeed, it is arguably the atomistic view of the human being as self-realizing and self-interested that is the aberration, a conception relegated to the sidelines in terms of its practical impact on social organization until the rise of urbanism, with its tendency to normalize anonymity and impersonal relations (Hunter, 2018). Yet the human capacity to see oneself not only as a contributor to the wider moral project of a community, but as a moral project in one's own right, is not extinguished when humans find themselves in situations—colonized lifeworlds—that do not elicit such a vision. It remains, dormant perhaps, but more often active and vital to individual self-conceptions, albeit largely in non-political aspects of life. It is perhaps in opening the imagination of individuals, communities, and institutions to the applicability of this vision for human life to the political that an example such as the Bahá'í community has the potential to make the most notable contribution to our political culture. And in so doing, it might help illuminate the kinds of transformative changes that could allow humanity to advance toward the aspirations articulated in Goal 16.

## 7. Conclusion

Speaking of the need to pivot toward a socio-political order premised upon the unity of the human race, the Universal House of Justice writes:

To choose such a course is not to deny humanity's past but to understand it. The Bahá'í Faith regards the current world confusion and calamitous condition in human affairs as a natural phase in an organic process leading ultimately and irresistibly to the unification of the human race in a single social order whose boundaries are those of the planet. The human race, as a distinct, organic unit, has passed through evolutionary stages analogous to the stages of infancy and childhood in the lives of its individual members, and is now in the culminating period of its turbulent adolescence approaching its long-awaited coming of age (Universal House of Justice, 1985).

This paper has sought to highlight the ways in which the model of the Bahá'í community, in its efforts to prefigure the features of humanity's “coming of age,” may provide insight into how a socio-political order can not only resist the dynamic of modern politics in which system colonizes lifeworld, but infuse the relationship between the two with norms, practices, and structures that foster and protect communicative action as the principal mode of organization. I have attempted to show that the structural dynamics that lend a sense of inevitability to Habermas' historical analysis are catalyzed by particular ideas, whose contingency is highlighted by prefigurative efforts such as those of the Bahá'í community. I have further argued that this example may hold promise as a source of inspiration for designing an institutional system that is culturally and procedurally democratic, effective, accountable, and inclusive. Those laboring to reform existing institutions at all levels, as well as those attempting to design new institutions capable of meeting the unprecedented challenges facing the human family today, may find in examples such as these a source of insight worth exploring.

## Data availability statement

The original contributions presented in the study are included in the article/supplementary material, further inquiries can be directed to the corresponding author.

## Author contributions

The author confirms being the sole contributor of this work and has approved it for publication.

## Footnotes

^1^Simultaneously, our cognitive biases alert us to evidence that confirms our story about human beings, and blind us to evidence that suggests its contingency (see Bergman, 2020). As will be seen, prefigurative practice, which begins by imagining a different world, allows a community to see through different eyes.

^2^This diversity, which is a normative good actively pursued, means that Bahá'í attempts to construct a socio-political reality infused by communicative action do not occur in the context of a socio-economic monoculture, as was (relatively) the case for the bourgeois public sphere.

^3^The Bahá'í community also has a non-prefigurative facet: fulsome engagement with wider society—though not on all terms—is encouraged. While such engagement aims to find resonance and build relationships with like-minded individuals, communities and institutions, with a view to contributing to improvements in wider society, there simultaneously remains a distinctly prefigurative core to Bahá'í community life in the form of its own administration, described as the embryonic form of a “unique… wondrous System” without precedent (Bahá'u'lláh, 1994; *Kitáb-i-Aqdas* para. 181 and note 189). Community building, a distinct focus of the Bahá'í community, also has a prefigurative dimension but will not be the focus of this paper.

^4^Bahá'ís might find assertions about “the” Bahá'í position on some questions jarring, as they could be seen as implying authoritative interpretation, which is a centralized function in the Bahá'í Faith (Heller, 2022). In recognition of this, I shall acknowledge at the outset that what I am suggesting here is more accurately described as “a”, rather than “the”, Bahá'í position.

^5^Herbert Spencer was one of the first social theorists to fully articulate an organismic analogy of society. Spencer's heuristic model encourages one to focus on the interdependence between social systems and how they maintain equilibrium during structural change and social differentiation. In addition, attention is given to how institutions succeed or fail in developing and promoting the necessary social needs for optimal group functioning and overall societal survival. Many systems theorists have since drawn heavily on Spencer's work, but due to his widely unpopular and unsubstantiated Social Darwinism, he tends to receive little acknowledgement for his groundbreaking insights into systems analysis (see Turner, 1985). Furthermore, the few neo-functionalists who do recognize Spencer's contribution to the field, distance themselves not only from his “survival of the fittest” discourse, but also a utilitarian philosophy and hyper individualism that overemphasizes the benefits of the free-market economy while turning a blind eye to its deleterious effects on community well-being (Alexander, 1987, p. 199) – ideas that diametrically oppose Saadi's (or Bahá'u'lláh's) emphasis on human solidarity. That Bahá'u'lláh's human body metaphor both captures the humanist concern for all people of the Persian poet and resonates with the functionalist analysis of the Victorian sociologist suggests something of the nuance and multidimensionality of his thinking.

^6^A fourth criteria, basic comprehensibility, is sometimes included.

^7^Since this paper is concerned with the Bahá'í community as an example of a prefigurative community, it sets aside the question of how this formal Administrative Order interacts with political authorities in the wider community. In brief, these relationships are informed by the core prefigurative premise that means must be coherent with ends. Since the ends the Bahá'í Faith seeks to advance are centered around the unity of humankind, along the lines discussed earlier, it engages with wider society, including political authorities, in ways that do not involve partisanship, but will seek to advance questions of common concern with receptive groups and institutions. The question of the extent to which this kind of interaction succeeds in effecting desired changes in wider society is one for future research.

^8^Membership on this body is restricted to men. Bahá'í writings explicitly state that the reason for this, which is not currently known, will become clear in the future, but that it does not rest on any inequality or difference in ability between men and women (see Smith T., 2022).

^9^There should in fact be no way for an Assembly member to know who voted for them; “constituencies” should not exist.
